# Drug interaction potential of high-dose rifampicin in patients with pulmonary tuberculosis

**DOI:** 10.1128/aac.00683-23

**Published:** 2023-09-28

**Authors:** Ralf Stemkens, Veronique de Jager, Rodney Dawson, Andreas H. Diacon, Kim Narunsky, Sherman D. Padayachee, Martin J. Boeree, Stijn W. van Beek, Angela Colbers, Marieke J. H. Coenen, Elin M. Svensson, Uwe Fuhr, Patrick P. J. Phillips, Lindsey H. M. te Brake, Rob E. Aarnoutse, on behalf of the PanACEA consortium

**Affiliations:** 1 Department of Pharmacy, Research Institute for Medical Innovation, Radboud University Medical Center, Nijmegen, The Netherlands; 2 TASK, Cape Town, South Africa; 3 Division of Pulmonology and Department of Medicine, University of Cape Town and University of Cape Town Lung Institute, Cape Town, South Africa; 4 Department of Pulmonary Diseases, Research Institute for Medical Innovation, Radboud University Medical Center, Nijmegen, The Netherlands; 5 Department of Clinical Chemistry, Erasmus University Medical Center, Rotterdam, The Netherlands; 6 Department of Pharmacy, Uppsala University, Uppsala, Sweden; ^7^ Clinical Pharmacology, Department I of Pharmacology, Center for Pharmacology, Faculty of Medicine and University Hospital Cologne, University of Cologne, Cologne, Germany; 8 UCSF Center for Tuberculosis, University of California, San Francisco, California, USA; Bill & Melinda Gates Medical Research Institute, Cambridge, Massachusetts, USA

**Keywords:** tuberculosis, high-dose rifampicin, drug interactions, metabolic phenotyping

## Abstract

Accumulating evidence supports the use of higher doses of rifampicin for tuberculosis (TB) treatment. Rifampicin is a potent inducer of metabolic enzymes and drug transporters, resulting in clinically relevant drug interactions. To assess the drug interaction potential of higher doses of rifampicin, we compared the effect of high-dose rifampicin (40 mg/kg daily, RIF40) and standard-dose rifampicin (10 mg/kg daily, RIF10) on the activities of major cytochrome P450 (CYP) enzymes and P-glycoprotein (P-gp). In this open-label, single-arm, two-period, fixed-order phenotyping cocktail study, adult participants with pulmonary TB received RIF10 (days 1–15), followed by RIF40 (days 16–30). A single dose of selective substrates (probe drugs) was administered orally on days 15 and 30: caffeine (CYP1A2), tolbutamide (CYP2C9), omeprazole (CYP2C19), dextromethorphan (CYP2D6), midazolam (CYP3A), and digoxin (P-gp). Intensive pharmacokinetic blood sampling was performed over 24 hours after probe drug intake. In all, 25 participants completed the study. Geometric mean ratios (90% confidence interval) of the total exposure (area under the concentration versus time curve, RIF40 versus RIF10) for each of the probe drugs were as follows: caffeine, 105% (96%–115%); tolbutamide, 80% (74%–86%); omeprazole, 55% (47%–65%); dextromethorphan, 77% (68%–86%); midazolam, 62% (49%–78%), and 117% (105%–130%) for digoxin. In summary, high-dose rifampicin resulted in no additional effect on CYP1A2, mild additional induction of CYP2C9, CYP2C19, CYP2D6, and CYP3A, and marginal inhibition of P-gp. Existing recommendations on managing drug interactions with rifampicin can remain unchanged for the majority of co-administered drugs when using high-dose rifampicin. Clinical Trials registration number NCT04525235.

## INTRODUCTION

Rifampicin is the cornerstone of the treatment of drug-sensitive tuberculosis (TB), a disease that remains a major global health problem with 10.6 million new cases and 1.6 million deaths worldwide in 2021 (1). The standard dose of rifampicin has been 10 mg/kg/day since its approval in 1971 by the US Food and Drug Administration (2). A growing body of evidence supports the use of higher doses of rifampicin. Doses up to 40 mg/kg in adults have yielded improved early bactericidal activity, may shorten TB treatment, and are well tolerated (3 – 6). In addition, higher doses of rifampicin may reduce mortality in patients with TB meningitis (7, 8). Some TB referral centers already use higher doses of rifampicin in clinical practice (9). Many clinical trials in both adults and children are currently evaluating high-dose rifampicin in the treatment of active TB and latent TB infection.

Rifampicin is notorious for its capacity to cause drug interactions as it is a potent inducer of several metabolic enzymes and drug transporter proteins (10). It decreases exposure to many co-administered drugs, including anti-retroviral, anti-diabetic, and cardiovascular drugs (10). Little is known about the maximal inductive capacity of rifampicin. Some small studies suggest that maximal induction already occurs at lower rifampicin doses of 300–600 mg daily, but these studies only evaluated the effect of rifampicin on selected drugs and provided no data on higher rifampicin doses (30–40 mg/kg) (11 – 13). High-dose rifampicin (35 mg/kg) does not affect the exposure to isoniazid, pyrazinamide, and ethambutol, compared to standard-dose rifampicin (5). In a recent study, patients receiving high-dose rifampicin (35 mg/kg) exhibited reduced plasma exposures to the anti-retroviral drugs dolutegravir and efavirenz as compared to patients receiving standard-dose rifampicin (14). However, the drug interaction potential of high rifampicin doses remains unknown for all other drugs.

Phenotyping for drug-metabolizing enzymes or transporters is deﬁned as measuring the actual *in vivo* activity in an individual. This is performed by a single administration of a selective substrate for an enzyme or transporter (probe drug) and subsequent determination of a phenotyping metric, preferably the total exposure to the probe drug. Multiple probe drugs can be applied simultaneously as a “cocktail” to assess the activity of metabolic enzymes and transporters at the same time (15, 16). We conducted a phenotyping cocktail study in participants with pulmonary TB to assess the effect of optimized, high-dose rifampicin (40 mg/kg/day; RIF40), as compared to a standard dose of 10 mg/kg/day (RIF10), on the activity of five major cytochrome P450 (CYP) enzymes [CYP1A2, CYP2C9, CYP2C19, CYP2D6, and CYP3A4/5 (CYP3A)] and P-glycoprotein (P-gp). Many commonly prescribed drugs are substrates of these enzymes (17).

## RESULTS

### Study population

A total of 38 participants were screened, of whom 30 participants were enrolled in the study (see Fig. S1). Five participants withdrew before study completion, four due to a positive COVID-19 test, and one participant due to adverse events (AEs; headache, nausea, and flushing after commencing RIF40). In all, 25 participants completed the study and were included in the demographic, safety, and pharmacokinetic (PK) analyses. Patient characteristics are shown in Table 1.

**TABLE 1:** Demographic and baseline characteristics

*N*	25
Age, years, median (range)	28 (21–47)
Body weight, kg, median (range)	56 (43–79)
Male sex, *n* (%)	14 (56)
Race, *n* (%)	
Black	12 (48)
Colored	13 (52)
Smoking status, *n* (%)	
Smoker	13 (52)
TB treatment duration before the study, days, median (range)	84 (63–123)

### Safety and adherence to treatment

A total of 35 AEs were reported for 15 participants: 24 grade 1 (*n* = 14 participants), 5 grade 2 (*n* = 4 participants), and 6 grade 3 (*n* = 4 participants). Two of the ≥grade 2 AEs were deemed definitely related to the study medication, and 6 of the ≥grade 2 AEs occurred in the same participant (see Table S7). In general, the study medication was well tolerated. Adherence to TB treatment was high and, according to pill count, ranged from 93% to 100%.

### Effect of RIF40 on the phenotyping metrics of the probe drugs

The geometric mean (GM) area under the curve (AUC)_0–24 h_ of rifampicin with RIF40 was 318.9 mg/L*h, ∼eightfold higher than with RIF10. This greater than dose proportional increase in exposure is consistent with the well-known non-linear pharmacokinetics of rifampicin (3, 5). The GM AUC_0–24h_ of isoniazid was similar at both PK sampling days (Table 2). Rifampicin and isoniazid exposures corresponded well with reported data but were somewhat higher in this study (4).

**TABLE 2:** Rifampicin and isoniazid pharmacokinetics during treatment with RIF10 (day 15) and RIF40 (day 30)^*a*^

PK parameter	RIF10 (day 15)	RIF40 (day 30)
	*n* = 25	*n* = 25
Rifampicin AUC_0–24 h_ (mg/L*h)	39.4 (34.9–44.5)	318.9 (286.2–355.4)
Rifampicin *C*_max_ (mg/L)	7.6 (7.0–8.4)	43.2 (40.6–45.9)
Isoniazid AUC_0–24 h_ (mg/L*h)	10.8 (8.6–13.4)	10.9 (8.7–13.7)
Isoniazid *C*_max_ (mg/L)	2.5 (2.2–2.8)	2.5 (2.2–2.8)

^*a*^ PK parameters are depicted as geometric mean (95% CI).

**Fig 1:**
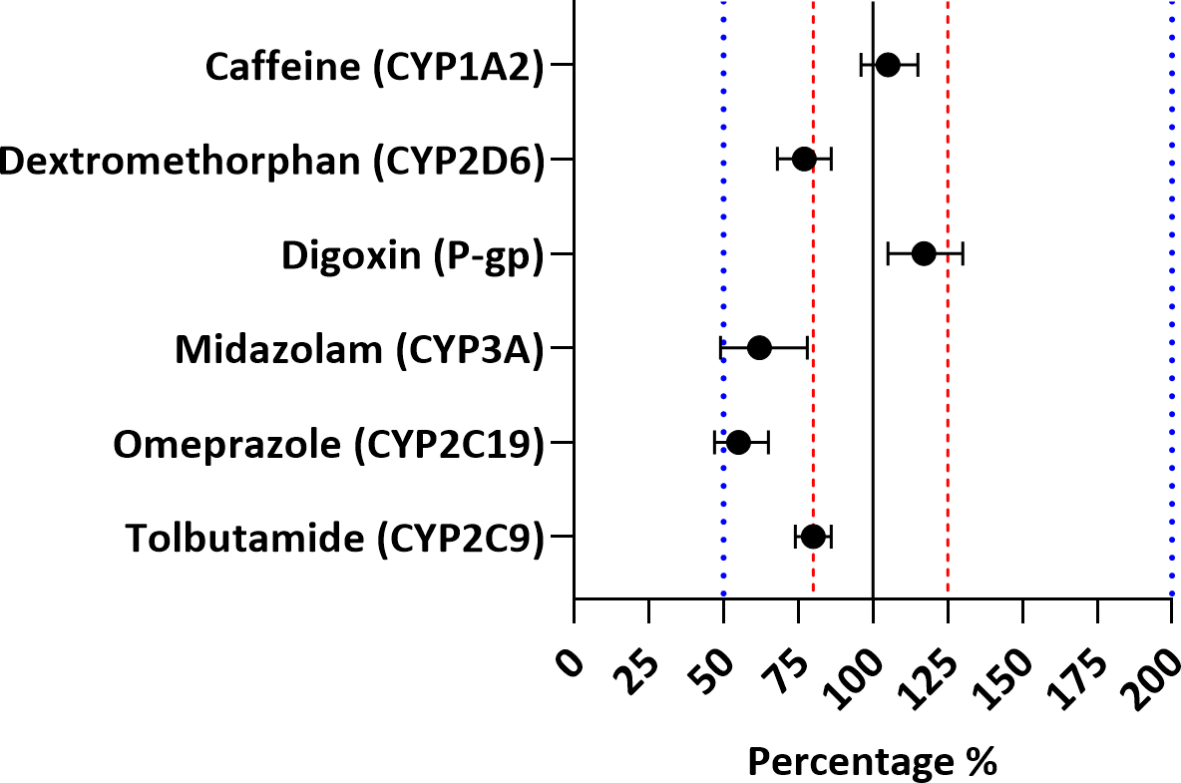
Geometric mean ratios (%) of AUC values (RIF40 versus RIF10) with 90% CI for all the probe drugs. The solid line is the unity line (i.e., no difference between RIF10 and RIF40). The dashed lines represent the standard bioequivalence range of 80%–125% (20). GMR estimates with 90% CI entirely within this range were considered to indicate no significant additional interaction with RIF40. The dotted lines represent the range that indicates a mild additional interaction (≤twofold decrease or increase) (18, 19).

The geometric mean ratio (GMR) estimates of all phenotyping metrics (RIF40 versus RIF10) are depicted in Fig. 1; Table 3. The GMR of the AUC_0–∞_ with 90% confidence interval (CI) for caffeine (105%; 90% CI: 96–115%) was within the standard bioequivalence range of 80%–125%, whereas this range was exceeded for all other probe drugs (Table 3). The GMR (90% CI) for tolbutamide (CYP2C9), dextromethorphan (CYP2D6), and midazolam (CYP3A) were 80% (74%–86%), 77% (68%–86%), and 62% (49%–78%), respectively. The GMR (90% CI) of the AUC_0–24 h_ and *C*_max_ for digoxin (P-gp) were 117% (105%–130%) and 117% (102%–135%). Due to the atypical PK profiles of omeprazole, the AUC_0–∞_ could often not be calculated. The AUC until the last measurable concentration (AUC_0–last_) was used as an alternative. The GMR (90% CI) of this AUC_0–last_ was 55% (47%–65%). A detailed overview of the PK parameters of the probe drugs is depicted in Table S8. Based on these findings, the additional interaction caused by high-dose rifampicin was classified as absent (CYP1A2) or mild (CYP2C9, CYP2C19, CYP2D6, CYP3A, and P-gp) (18, 19).

**TABLE 3:** Primary phenotyping metrics of the probe drugs with RIF10 and RIF40

Primary phenotyping metrics	RIF10 (day 15)	RIF40 (day 30)	GM ratio RIF40/RIF10 % (90% CI)	90% CI within 80%–125%
	Geometric mean h*ug/L (95% CI)			
	*n* = 25			
Caffeine AUC_0–∞_ (CYP1A2)	20,474 (16,238–25,814)	21,574 (16,548–28,126)	105 (96–115)	Yes
Tolbutamide AUC_0–∞_ (CYP2C9)	88,623 (75,014–104,701)	70,722 (56,742–88,147)	80 (74–86)	No
Omeprazole AUC_0–last_ (CYP2C19)	55.7 (39.1–79.3)	30.8 (21.3–44.4)	55 (47–65)	No
Dextromethorphan AUC_0–∞_ (CYP2D6)	14.7^*a*^ (9.2–23.5)	12.0^*a*^ (7.3–19.8)	77 (68–86)	No
Midazolam AUC_0–∞_ (CYP3A)	7.1^*b*^ (5.2–9.6)	4.4^*b*^ (3.4–5.9)	62 (49–78)	No
Digoxin AUC_0–24 h_ (P-gp)	8.6^*c*^ (7.4–10.0)	10.1^*c*^ (8.9–11.4)	117 (105–130)	No

^*a*^ *N* = 21 and *N* = 19 with RIF10 and RIF40, respectively; AUC_0–∞_ of dextromethorphan could not be estimated reliably in all participants, because the percentage extrapolated was ≥20%. Of note, the AUC_0–last_ was calculated as an alternative metric for all participants and yielded similar results; GMR (90% CI) was 69% (61%–79%).

^*b*^ *N* = 24; AUC_0–∞_ of midazolam could not be estimated in one participant due to insufficient data points for extrapolation.

^*c*^ *N* = 24; one exclusion due to a decreased renal function (an increase of ≥1.5 times the serum creatinine concentration between day 15 and day 30).

### Subgroup analyses

Caffeine concentrations were 55%–60% lower among smokers, regardless of rifampicin dose (Table S9). The GMR estimate of the AUC_0–∞_ for caffeine was similar for smokers (*n* = 13) and non-smokers (*n* = 12), namely 100% (87%–114%) and 112% (98%–128%). Genetic evaluation (Table S10) revealed two participants with possibly reduced activity of CYP1A2 and one poor metabolizer (PM) of CYP2C19. The GMRs of caffeine and omeprazole without these participants were 106% (96%–118%) and 56% (48%–67%), which were similar to the results in the total study population.

## DISCUSSION

This study evaluated the drug interaction potential of high-dose rifampicin in a target population of TB patients, using metabolic phenotyping as an efficient instrument to screen for multiple drug interactions simultaneously. The results demonstrate that high-dose rifampicin (40 mg/kg daily) has no additional effect on the activity of CYP1A2, caused mild additional induction (≤50% reduction in exposure to probe drugs) of CYP2C9, CYP2C19, CYP2D6, and CYP3A, and slightly inhibited P-gp, compared to standard-dose rifampicin (10 mg/kg daily). This infers that these effects will have no clinical implication for the majority of drugs that are co-administered with high-dose rifampicin in clinical practice and clinical trials across the world.

**Fig 2:**
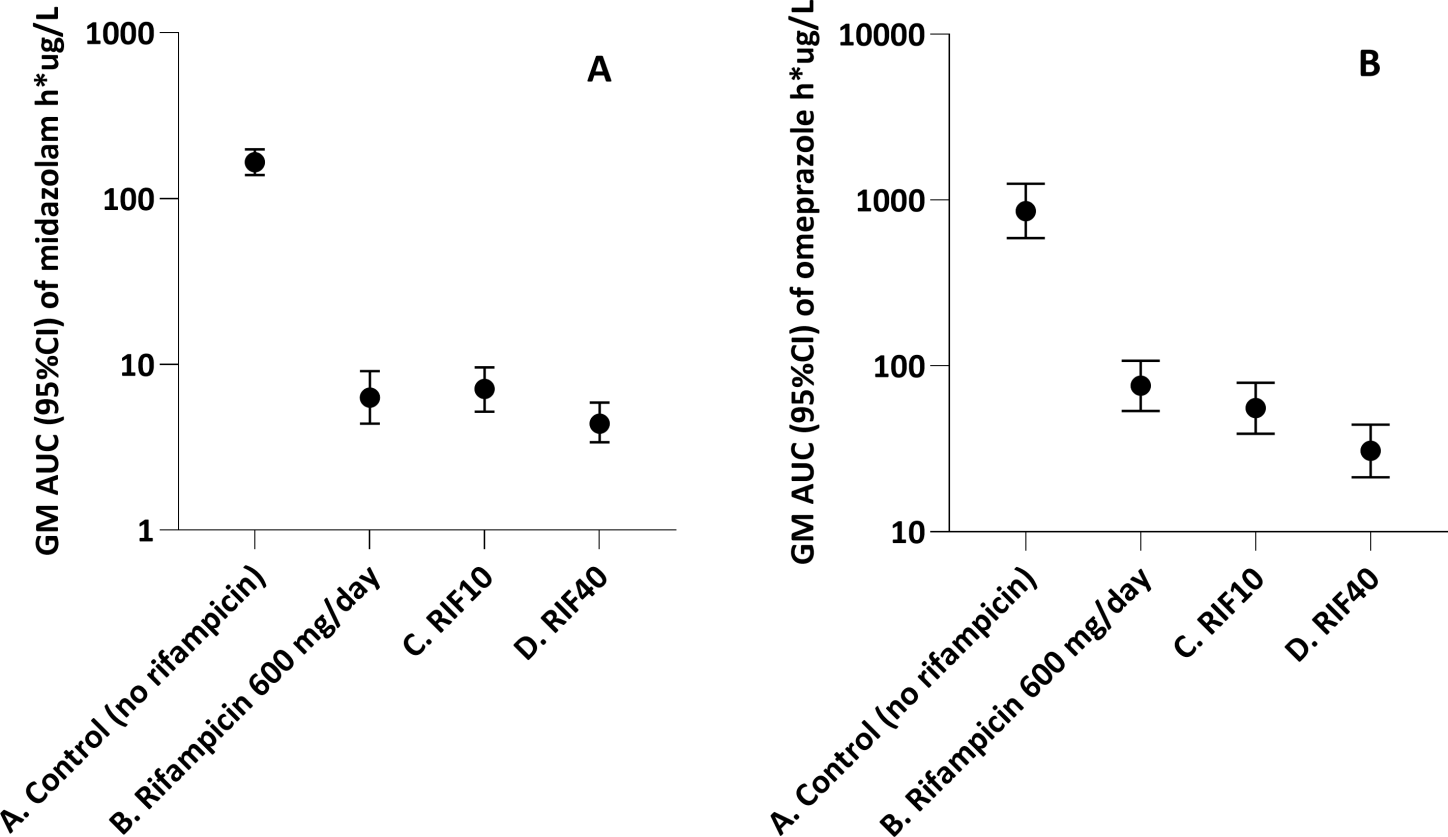
Comparison of midazolam and omeprazole PK with reference studies (with and without rifampicin). Figure A depicts GM AUC_0–∞_ values (95% CI) of midazolam after an oral dose of 15 mg in healthy volunteers without and with rifampicin (**A and B**), as well as with RIF10 and RIF40 in our study (**C and D**). Figure B shows GM AUC values (95% CI) of omeprazole after an oral dose of 20 mg in healthy volunteers (AUC_0–∞_) without and with rifampicin (**A and B**), as well as with RIF10 and RIF40 in our study (AUC_0–last,_ **C and D**). The AUC values from the reference studies were converted to h*ug/L for this figure (21, 22).

The largest effects in this study were observed for midazolam (CYP3A) and omeprazole (CYP2C19). RIF40 reduced midazolam and omeprazole exposures by 38% and 45%. The additional induction of CYP3A and CYP2C19 as compared to RIF10 would formally be classified as mild, while the width of the 90% CIs details that a borderline moderate additional interaction (>50% reduction in exposure) cannot be excluded (18, 19). It is important to emphasize that these reductions are much smaller than the effect of standard-dose rifampicin (versus no rifampicin) on exposures to midazolam and omeprazole. More specifically, it has been shown that rifampicin (600 mg daily) versus no rifampicin causes ∼96% (∼20-fold) and ∼90% (∼10-fold) reductions in midazolam and omeprazole exposures, respectively (21, 22), see Fig. 2. Therefore, the observed additional induction is not expected to be clinically relevant for the majority of CYP3A and CYP2C19 substrates. It has no implications for CYP3A or CYP2C19 substrates that are already contra-indicated during treatment with RIF10 (e.g., direct oral anticoagulants). Additional induction is also not expected to have clinical implications for most co-administered drugs that require dose titration based on clinical effect when administered with rifampicin (e.g., calcium channel blockers). For drugs with a narrow therapeutic index, additional care may, however, be warranted when using RIF40. Additional induction may also be relevant for orally administered CYP3A substrates with fixed dosing recommendations (e.g., dexamethasone for TB meningitis, where examples for CYP2C19 are lacking) (23).

Of pharmacological interest, the decreased AUC_0–∞_ of midazolam with RIF40 in this study corresponded with a decreased peak plasma concentration (4.2 versus 2.6 µg/L), whereas the elimination half-life was similar with RIF10 and RIF40 (1.3 and 1.2 hours). This suggests that additional induction of CYP3A by RIF40 relates to first-pass metabolism, corresponding with previous studies (24, 25), and is probably most relevant to orally administered drugs.

Atypical PK profiles were observed for omeprazole. One possible explanation for this observed PK behavior is its enteric-coated formulation, which may have led to irregular absorption of omeprazole (16). Consequently, results for omeprazole (CYP2C19) should be interpreted with caution. Furthermore, omeprazole is primarily metabolized by CYP2C19, but CYP3A4 also plays a role in its metabolism. The reduction in exposure to RIF40 may not be solely attributable to CYP2C19 but also CYP3A4 (16). RIF40 also caused a reduction of 23% in dextromethorphan AUC_0–∞_.

Dextromethorphan is predominantly metabolized via CYP2D6, but CYP3A4 is also involved in its metabolism (26). Overall, the mild additional induction, whether (solely) attributable to CYP2D6 or not, is not expected to be clinically relevant.

A small decrease in tolbutamide AUC_0–∞_ (20%) was observed with RIF40. It has previously been shown that a standard dose of rifampicin reduces the AUC_0–∞_ of tolbutamide by 65% (27). The mild additional induction of CYP2C9 by RIF40 is not expected to be clinically relevant.

No differences in caffeine AUC_0–∞_ were observed between RIF40 and RIF10, indicating no additional induction of CYP1A2. These results were similar for smokers and non-smokers which suggests that smoking, known to cause induction of CYP1A2, did not affect the induction potential of RIF40 for this enzyme (28).

RIF40 was associated with a slightly increased digoxin AUC_0–24 h_ and *C*_max_ (both 17%), which is not deemed clinically relevant. In contrast, previous work has shown that standard-dose rifampicin decreased the digoxin AUC_0–144 h_ by 30% as a result of P-gp induction (29). However, as rifampicin is also known to inhibit intestinal P-gp, this minimal increase in AUC_0–24 h_ could be due to additional P-gp inhibition by RIF40, outweighing possible additional induction of P-gp (30). The extent of this inhibitory effect may be dependent on the interval between digoxin and rifampicin administration (30). The possibility of a stronger inhibitory effect during simultaneous administration cannot be excluded.

There are minimal data on the maximal inductive capacity of rifampicin. It has been long suspected that this induction capacity was maximal with standard rifampicin doses (11 – 13). A recent study has shown that a high dose of rifampicin (35 mg/kg daily) reduced the trough concentrations of dolutegravir (a substrate of UDP glucuronosyltransferase (UGT) 1A1 and CYP3A4) and mid-dose concentrations of efavirenz (a substrate of CYP2B6) by 43% and 37%, as compared to 10 mg/kg rifampicin (14). No loss of virological control of HIV was observed in participants with dolutegravir or efavirenz concentrations below target thresholds, but the study was not powered to assess virologic efficacy (14). Overall, these data correspond well with our results, showing some additional induction with higher doses of rifampicin.

This study had some limitations. First, the study did not include a control arm without rifampicin which would have provided a more complete picture of the effect of increasing rifampicin doses versus no rifampicin. However, the drug interaction potential of standard-dose rifampicin is well established, and therefore, our results can be evaluated in perspective (see Fig. 2). Second, we assessed the effect of (high dose) rifampicin in the presence of isoniazid, which is an inhibitor of CYP2C19 and CYP3A4 (31). However, we believe that this does not impact our results as the potent inductive effect of rifampicin outweighs the inhibitory effect of isoniazid, and the same dose of isoniazid was used throughout the study. Finally, the effect of RIF40 on other metabolic enzymes (e.g., CYP2B6, UGT) and transporters was not evaluated in this study. It is however well known that CYP3A4 is most susceptible to rifampicin induction, and the additional effects on other enzymes are not expected to exceed those on CYP3A4 (10). In addition, many commonly prescribed drugs are substrates of the investigated CYP enzymes and P-gp (10, 17, 32).

In conclusion, high-dose rifampicin results in no additional effect on the activity of CYP1A2, shows mild additional induction of CYP3A, CYP2C19 and, to a lesser extent, CYP2C9 and CYP2D6, while slightly inhibiting P-gp. These results indicate that clinicians can use existing recommendations on managing interactions with standard-dose rifampicin for the majority of co-administered drugs when using high-dose rifampicin. Further interaction studies may be warranted, focusing on high-dose rifampicin and CYP3A or CYP2C19 substrates with a narrow therapeutic index.

## MATERIALS AND METHODS

### Study population

Adults (18–65 years) with drug-sensitive pulmonary TB, in the continuation phase of standard TB treatment, using a standard dose of 10 mg/kg rifampicin and isoniazid, were included in this study (see Table S1 for inclusion and exclusion criteria). Written informed consent to participate in the trial was obtained from all participants.

### Study design

This open-label, single-arm, two-period, fixed-order phenotyping cocktail study was performed at two study sites, TASK Clinical Research Center and the University of Cape Town Lung Institute, in Cape Town, South Africa. The study protocol was approved by local ethical review boards and by the South African Health Products Regulatory Authority (SAHPRA) and was conducted according to Good Clinical Practice standards. This trial is registered with ClinicalTrials.gov as trial number NCT04525235.

**Fig 3:**
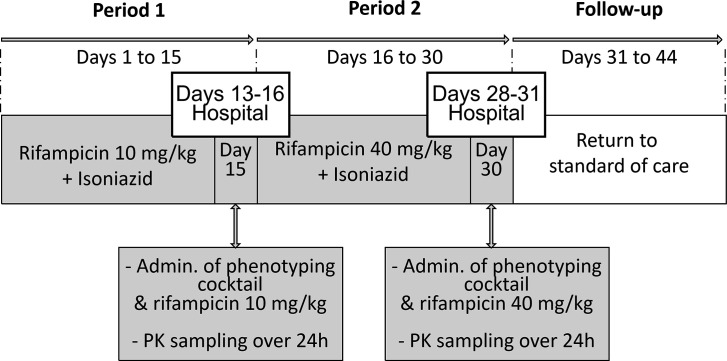
Schematic overview of the study design.

All participants received RIF10 (i.e., a continuation of the dose as per standard care) in a fixed combination with isoniazid (Rifinah) for 15 days (period 1), followed by RIF40 [Rifinah + additional loose rifampicin (Rifadin) capsules] for 15 days (period 2, see Fig. 3 for a schematic overview). The duration of treatment with RIF40 was selected to achieve maximal (additional) induction of CYP enzymes and P-gp after increasing the dose of rifampicin (10). During period 2, all TB drugs were administered with breakfast to prevent or alleviate possible adverse effects related to high-dose rifampicin. Dosing was weight banded (Tables S2 and S3). Adherence to TB therapy was evaluated using treatment registration cards, self-assessment by participants, and pill counts. Participants were hospitalized during the last 3 days of each study period. On day 15 of each study period, a phenotyping cocktail consisting of six probe drugs was administered. Participants received a single oral dose of caffeine (150 mg), tolbutamide (125 mg), omeprazole (20 mg), dextromethorphan (30 mg), midazolam (15 mg), and digoxin (0.5 mg) to assess the activity of CYP1A2, CYP2C9, CYP2C19, CYP2D6, CYP3A, and P-gp, respectively. Table S4 shows details on the composition of the phenotyping cocktail. This cocktail was based on the well-established Cologne cocktail (15, 16, 33). The probe drugs were administered on an empty stomach following overnight fasting (of at least 8 hours duration), and participants remained fasted until 4 hours following probe drug administration. Rifampicin and isoniazid were administered 4 hours after administration of the probe drugs, with a standardized meal. This time interval was chosen to prevent a food effect on the PK of the probe drugs and to minimize any inhibitory effects of rifampicin on enzyme or transporter activities, which might mask additional inductive effects (30, 34).

### Monitoring of safety and concomitant medications

Participants were closely monitored throughout the study for evidence of clinical- or laboratory-based AEs. Grading and classification of AEs occurred according to the Common Terminology Criteria for Adverse Events (CTCAE, v 5.0) (35). All concomitant medications (and changes thereof) were reviewed for their inductive or inhibitory potential as well as to identify possible substrates of CYP enzymes that could potentially be affected by additional induction with RIF40.

### Pharmacokinetic and pharmacogenetic blood sampling and bioanalysis

Blood samples for assessment of PK parameters of the phenotyping cocktail and the TB drugs were collected on day 15 and day 30. Samples for PK assessment of each of the probe drugs were drawn pre-dose and at 0.5, 1, 1.5, 2, 3, 4, 5, 6, 8, 10, 12, and 24 hours post-dose. Blood samples for PK assessment of the TB drugs were drawn pre-dose and at 2, 4, and 6 hours after rifampicin and isoniazid intake. In addition, a blood sample was collected for pharmacogenetic testing to identify the genotypes of the CYP enzymes.

Total (protein-bound plus unbound) concentrations of the probe drugs and rifampicin/isoniazid in plasma were measured with validated liquid chromatography-mass spectrometry methods at Nuvisan (Neu-Ulm, Germany) and Radboudumc (Nijmegen, the Netherlands), respectively. Details on the PK and pharmacogenetic assays are described in supplementary texts S1, S2 and Tables S5 and S6.

### Pharmacokinetic analysis

Non-compartmental pharmacokinetic analysis was conducted using Phoenix WinNonlin v6.4 (Certara USA Inc., Princeton, NJ, USA) to determine the phenotyping metrics [AUC_0–∞_ or until 24 hours (AUC_0–24 h_) for digoxin because of its long elimination half-life] and other PK parameters of each of the probe drugs when combined with RIF10 (day 15) and RIF40 (day 30). For digoxin, the peak concentration (*C*_max_) was assessed as a secondary phenotyping metric, specifically to assess intestinal P-gp activity. Individual PK parameters of rifampicin and isoniazid were estimated with established population PK models, based on relevant individual characteristics, dosing information, and observed drug concentrations, using NONMEM software (36 – 38).

### Statistical analyses

Based on reported data on intrasubject variability in pharmacokinetic data of probe drugs, a sample size of 25 participants was assessed (33, 39, 40). Participant demographics and study outcomes were reported for participants who completed the study. The effect of RIF40 in comparison to RIF10 on the phenotyping metrics of the different probe drugs was evaluated with a mixed-model bio-equivalence analysis, using Phoenix WinNonlin. The main pharmacokinetic parameter under evaluation was the AUC, for which a GMR of all the probe drugs was calculated (RIF40 versus RIF10). GMR estimates with a 90% CI entirely within the range of 80%–125% were considered to indicate no significant additional interaction (20). The extent of additional induction was classified as mild, moderate, and strong in case of ≤50%, >50 to ≤80%, and >80% reductions in AUC, respectively, based on guidelines for drug interactions (18, 19). A subgroup bio-equivalence analysis was performed for caffeine to separate smokers from non-smokers, as smoking can cause CYP1A2 induction (28). In addition, a subgroup analysis excluding PMs (i.e., two non-functional alleles) or, in the case of CYP1A2, individuals with possibly reduced activity, was performed for all CYP enzymes.

Statistical analyses were performed using IBM SPSS Statistics for Windows (v.27.0 Armonk, NY: IBM Corp.)

## SUPPLEMENTAL MATERIAL

Supplementary material aac.00683-23-s0001.docx Supplementary figures, tables and texts

ASM does not own the copyrights to Supplemental Material that may be linked to, or accessed through, an article. The authors have granted ASM a non-exclusive, world-wide license to publish the Supplemental Material files. Please contact the corresponding author directly for reuse.
