# Supplementary material for: Drug interaction potential of high-dose rifampicin in patients with pulmonary tuberculosis
Source: Antimicrob Agents Chemother. 2023 Sep 28;67(10):e00683-23. doi: 10.1128/aac.00683-23 (PMC10583668; doi:10.1128/aac.00683-23)
Supplement: Supplementary material — Supplementary figures, tables and texts [file aac.00683-23-s0001.docx]

**Supplementary material

Overview of content**

**Methods
Table S1. In- and exclusion criteria
Table S2. Weight banded dosing of standard dose rifampicin (10 mg/kg) and isoniazid
Table S3. Weight banded dosing of high-dose rifampicin (40 mg/kg) and isoniazid
Table S4. Overview of the phenotyping cocktail
Text S1. Bioanalysis of probe drugs and anti-TB drugs
Table S5. Validation parameters of the bioanalytical assays for the probe drugs
Table S6. Validation parameters of the bioanalytical assays for rifampicin and isoniazid
Text S2. Genotyping of cytochrome P450 (CYP) genes

Results/discussion
Figure S1. Overview of study population
Table S7. Description of ≥ grade 2 adverse events (AEs)
Table S8. PK parameters of the probe drugs
Table S9. Subgroup analysis of the primary phenotyping metrics of caffeine for smokers and nonsmokers
Table S10. Overview of the CYP genotypes**

**Methods**

| **Table S1. In- and exclusion criteria** | |
| --- | --- |
| Inclusion criteria | |
| 1. | The participant is able and willing to provide written, informed consent prior to all trial-related procedures. |
| 2. | The participant is aged between 18 and 65 years, inclusive. |
| 3. | The participant is a diagnosed pulmonary TB patient. |
| 4. | The participant is currently being treated with a daily dose of 10 mg/kg rifampicin, i.e. 450 mg daily for patients with a body weight below 55 kg and 600 mg daily for participants with a body weight above 55 kg. This is in correspondence with the local South African TB treatment program. Furthermore, the participant has to be in the continuation phase of the treatment regimen (i.e. month 3 to 6), has demonstrated reasonable treatment compliance (≥80% of doses) and tolerates treatment well. |
| 5. | The participant has a body weight (in light clothing and with no shoes) between 40 and 85 kg, inclusive. |
| 6. | The participant is and stays non-pregnant (based on a negative serum pregnancy test,) and non-lactating (female participants of childbearing potential only). |
| Exclusion criteria | |
| 1. | The patient is in poor general condition where any change in treatment cannot be accepted per discretion of the Investigator. |
| 2. | The participant has active Hepatitis B. |
| 3. | The participant has active Hepatitis C. |
| 4. | The participant is receiving antiretroviral therapy (ART). |
| 5. | There is evidence showing the participant has clinically significant metabolic, gastrointestinal, or other abnormalities than could possibly alter the PK of rifampicin and/or the probe drugs. |
| 6. | The participant has a history of or current clinically relevant cardiovascular disorder such as: heart failure, atrioventricular (AV) block, arrhythmia, tachyarrhythmia or status after myocardial infarction. |
| 7. | The participant has a family history of sudden death of unknown or cardiac-related cause, or of prolonged QTc interval. |
| 8. | The participant has clinically relevant abnormalities in the ECG such as atrioventricular (AV) block, prolongation of the QRS complex over 100 milliseconds, or of a QTc interval over 450 milliseconds on the screening ECG. |
| 9. | The participant has abnormal alanine aminotransferase (ALT) and/or aspartate transferase (AST) levels > 3 times the upper limit of the laboratory reference range at screening. |
| 10. | The participant has a known or suspected, current drug or amphetamine abuse, that is, in the opinion of the Investigator, sufficient to compromise the safety or cooperation of the patient. |
| 11. | The participant used any drugs or substances known to be strong inhibitors or inducers of cytochrome P450 enzymes and/or P-glycoprotein (P-gp) within 2 weeks prior to day 1 (i.e. 1 month before administration of the phenotyping probes on day 15) of the study (including carbamazepine, barbiturates, St. John’s Wort, clarithromycin, itraconazole, fluconazole, quinidine, ketoconazole, erythromycin). Exceptions may be made for participants who have received 3 days or less of one of these drugs or substances, if there has been a wash-out period equivalent to at least 5 half-lives of that drug or substance before day 1 of the study. |
| 12. | The participant uses any of the phenotyping probe drugs (i.e. midazolam, caffeine, dextromethorphan, tolbutamide, omeprazole and digoxin) as part of standard medical treatment. |
| 13. | The participant has as history of allergy to any of the phenotyping probe drugs (i.e. midazolam, caffeine, dextromethorphan, tolbutamide, omeprazole and digoxin) |

| **Table S2. Weight banded dosing of standard dose rifampicin (10 mg/kg) and isoniazid** | | |
| --- | --- | --- |
| Bodyweight (kg) | Rifinah® fixed combination 150/75 mg | Rifinah® fixed combination 300/150 mg |
|  | Number of tablets | |
| 40-54 | 3 | 0 |
| ≥55 | 0 | 2 |

| **Table S3. Weight banded dosing of high-dose rifampicin (40 mg/kg) and isoniazid** | | | | |
| --- | --- | --- | --- | --- |
| Bodyweight (kg) | Rifinah® fixed combination 150/75 mg | Rifinah® fixed combination 300/150 mg | Capsules rifampicin (Rifadin®) 150 mg | **Capsules rifampicin 300 mg (Rifadin**®) |
|  | Number of tablets and capsules | | | |
| 40-54 | 3 | 0 | 1 | 4 |
| 55-70 | 0 | 2 | 0 | 6 |
| >70 | 0 | 2 | 1 | 8 |

| **Table S4. Overview of the phenotyping cocktail** | | | |
| --- | --- | --- | --- |
| Probe drugs | Formulation | Dose^a^ | Route of administration |
| Caffeine | Tablet 150mg (Regmakers®) | 150 mg  (= 1 tablet) | Oral |
| Tolbutamide | Tablet 500 mg (from Centrafarm BV) | 125 mg (= ¼ tablet^b^) | Oral |
| Omeprazole | Capsule 20 mg (Altosec®) | 20 mg (= 1 capsule) | Oral |
| Dextromethorphan | Syrup 3 mg/ml (Benylin®) | 30 mg  (= 10 ml tablet) | Oral |
| Midazolam | Tablet 15 mg (Dormicum®) | 15 mg (= 1 tablet) | Oral |
| Digoxin | Tablet 0.25 mg (Lanoxin®) | 0.5 mg (= 2 tablets) | Oral |

**^a^** The doses were based on studies performed with the Cologne cocktail [1, 2]. In our study we used a higher dose of midazolam, 15 mg instead of 2 mg as used in the Cologne cocktail. The reason was the strong induction of CYP3A4 by rifampicin which results in a (much) lower expected exposure to midazolam. A higher dose of midazolam was deemed justified and necessary in order to achieve plasma concentrations well above the lower limit of quantification to adequately describe the PK.
^b^  The tolbutamide tablets do not contain score lines for exact splitting in quarter tablets. The administered quarter (and total) tablets were weighed. A paired samples t-test showed that the calculated administered doses at day 15 were not significantly different from day 30; the mean difference was -1.6 % with a 95% CI of [-4.1% to 0.9%] (p = 0.2). Therefore, no dose corrections were applied for the assessment of the phenotyping metrics.

**Text S1. Bioanalysis of probe drugs and anti-TB drugs**

The intraday and interday accuracy for the probe drugs and rifampicin/isoniazid were 96-107% and 94-104%, respectively. The intraday and interday coefficients of variation (CVs) for the probe drugs and rifampicin/isoniazid were ≤8.8% and ≤6.1%, respectively. More details are shown in tables 5 and 6.

| **Table S5. Validation parameters of the bioanalytical assays for the probe drugs** | | | | | |
| --- | --- | --- | --- | --- | --- |
| Compound | Calibration range | Intraday | | Interday | |
|  | Concentration  (µg/L) | Accuracy  % | Precision  % | Accuracy  % | Precision  % |
| Caffeine | 20.0 - 5000 | 100 – 107 | 3.1 – 8.8 | 98 – 106 | 3.6 – 6.4 |
| Tolbutamide | 50.0 – 25000 | 100 – 104 | 3.7 – 7.4 | 104 – 107 | 5.3 – 7.6 |
| Omeprazole | 1.00 – 500 | 98 – 102 | 1.8 – 3.3 | 98 – 101 | 2.7 – 5.2 |
| Dextromethorphan | 0.05 – 25.0 | 97 – 100 | 1.7 – 2.5 | 96 – 99 | 2.4 – 4.3 |
| Midazolam | 0.15 – 75.0 | 99 – 106 | 0.8 – 3.4 | 97 – 102 | 2.2 – 4.4 |
| Digoxin | 0.02 – 10.0 | 99 – 103 | 3.9 – 7.8 | 99 – 102 | 3.4 – 8.8 |

| **Table S6. Validation parameters of the bioanalytical assays for rifampicin and isoniazid** | | | | | |
| --- | --- | --- | --- | --- | --- |
| Compound | Calibration range | Intraday | | Interday | |
|  | Concentration  (mg/L) | Accuracy  % | Precision  % | Accuracy  % | Precision  % |
| Rifampicin | 0.09-60.0 | 96 – 103 | 2.6 – 4.9 | 96 – 101 | 0.0 – 1.9 |
| Isoniazid | 0.045-15.0 | 94 – 104 | 3.2 – 6.1 | 95 – 103 | 0.0 – 0.8 |

**Text S2. Genotyping of cytochrome P450 (CYP) genes**

Genomic DNA was extracted automatically using the Chemagic DNA isolation kit special (PerkinElmer, Waltham, MA, USA) according to the manufacturer’s instructions.

Genotyping of the *cytochrome P450* (*CYP*) genes was performed using single-molecule molecular inversion probes as described previously at the Clinical Genetics department of the Maastricht University Medcial Center^+^ (MUMC^+^) [3]. smMIPs for *CYP1A2*, *CYP2B6*, *CYP2C9*, *CYP2C19*, *CYP2D6*, *CYP3A4* and *CYP3A5* were designed using the MIPgen pipeline [4]. smMIP library pools were prepared followed by sequencing on a NextSeq500 according to the manufacturer’s instructions. The following variants were assessed *1A, *1C, *1F, *1K, *3, *4, *6 and *7 in *CYP1A2*, *6 and *18 for *CYP2B6*, *2-*6, *8 and *11-*13 for *CYP2C9*, *2-*10 and *17 for *CYP2C19*, *2-*12, *14, *15, *17-*21, *29, *31, *33, *35, *36, *38, *40-*42 and the duplication for *CYP2D6*, *1A, *1B, *1G, *6, *8, *11, *13, *16-*18, *20, *22 and *26 in *CYP3A4* and *2-*7 in *CYP3A5*. The copy number variant (deletion/duplication) in *CYP2D6* was assessed using the LightCycler 480 II (Roche; LightCycler 480 Software release 1.5.1.62 SP3) according to the protocol described by Langaee et al [5]. Two probes were used; one in exon 9 and one in intron 6 Genetic variants and according phenotypes were called automatically using an in-house designed calling algorithm. Phenotype assignment was done based on the guidelines of the Dutch Pharmacogenetics Working Group (DPWG) [6]. If variants were not assigned by the DPWG, the phenotypes on PharmVar were followed [7]. The *1 allele assignment was used when the analyzed variants were not present in a patient.

**Results**


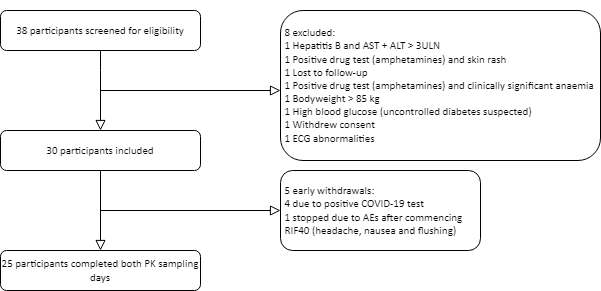


**Figure S1. Overview of study population.**

| **Table S7. Description of ≥ grade 2 adverse events (AEs)** | | | | |
| --- | --- | --- | --- | --- |
| Description AE | n | | CTCAE severity grade | Relatedness to study medication |
| **Participants with reported ≥ grade 2 AE, n (%)** | **5 (20%)** |  | |  |
| **≥ grade 2 AEs total** | **11** |  | |  |
| **AEs reported in period 1 (days 1-15)** | **3** |  | |  |
| Hypoalbuminaemina with proteinuria | 1^a^ | 3 | | Not related |
| Worsening of anemia | 1 | 3 | | Not related |
| Tinea capitis | 1^a^ | 2 | | Unlikely related |
| **AEs reported in period 2 (days 16-30)** | **8** |  | |  |
| Liver injury | 1 | 3 | | Definitely related |
| Neutropenia | 1 | 3 | | Possibly related |
| Worsening of anemia | 1^a^ | 3 | | Possibly related |
| Nephrotic syndrome | 1^ab^ | 3 | | Not related |
| Elevated ALT | 1 | 2 | | Unlikely related |
| HIV diagnosis | 1^a^ | 2 | | Not related |
| Impaired renal function | 1^a^ | 2 | | Not related |
| Allergic reaction | 1 | 2 | | Definitely related |
| ^a^ Six ≥ grade 2 AEs were reported for the same participant. ^b^ The nephrotic syndrome required hospitalization during follow-up, which was reported as a serious adverse event | | | | |
|  |  |  |  |  |

| **Table S8. PK parameters of the probe drugs** | | | | | | | | | | | | |
| --- | --- | --- | --- | --- | --- | --- | --- | --- | --- | --- | --- | --- |
|  | Caffeine (CYP1A2) | | Dextromethorphan (CYP2D6) | | Digoxin (P-gp) | | Midazolam (CYP3A4) | | Omeprazole (CYP2C19) | | Tolbutamide  (CYP2C9) | |
|  | N=25 | | N=25 | | N = 24^c^ | | N=25 | | N=25 | | N=25 | |
|  | RIF 10 mg/kg | RIF 40 mg/kg | RIF 10 mg/kg | RIF 40 mg/kg | RIF 10 mg/kg | RIF 40 mg/kg | RIF 10 mg/kg | RIF 40 mg/kg | RIF 10 mg/kg | RIF 40 mg/kg | RIF 10 mg/kg | RIF 40 mg/kg |
| C_max_ (ug/L) | 3296 (1810- 5030) | 3327 (2020 - 5360) | 1.30 (0.20-10.3) | 1.06 (0.12-15.9) | 0.74 (0.25-1.41) | 0.87 (0.46-1.42) | 4.2 (1.1-20.9) | 2.6 (0.5-14.7) | 39.1 (6.1-194) | 19.1 (3.6-59.6) | 12983 (6900-20800) | 11513 (2460-19100) |
| T_max_ (h) | 1.5 (0.5-3.0) | 1.6 (0.5-5.0) | 2.0 (1.0-5.0) | 2.0 (1.0-5.0) | 1.5 (0.5-6.0) | 4.0 (0.4-8.1) | 1.0 (0.5-2.1) | 0.5 (0.4-1.5) | 2.0 (0.5-5.0) | 2.0 (1.4-3.9) | 3.0 (1.0-5.0) | 2.1 (0.5-4.0) |
| AUC_0-24h_  (h*ug/L) | NA | | NA | | 8.6^d^ (3.1-16.4) | 10.1^d^ (4.6-15.9) | NA | | NA | | NA | |
| AUC_0-last_  (h*ug/L) | NA | | 10.3 (1.39-110) | 7.1 (0.60-130) | NA | | NA | | 55.7^f^ (8.1-427) | 30.8^f^ (3.4-142) | NA | |
| AUC_0-∞_ (h*ug/L) | 20474 (9989-75280) | 21574 (7468-75144) | 14.7^a^ (3.61-124) | 12.0^b^ (3.43-154) | NA | | 7.1 (1.7-28.2) | 4.4^e^ (1.5-23.8) | NA^f^ | | 88623 (28311-167684) | 70722 (9261-134742) |
| T½ (h) | 3.3 (2.1-8.0) | 3.5 (1.7-8.4) | 6.3^a^ (4.3-8.9) | 6.2^b^ (3.9-9.3) | NA | | 1.3 (0.7-5.8) | 1.2^e^ (0.6-3.5) | NA^f^ | | 3.8 (2.1-6.6) | 3.2 (1.1-6.0) |
| CL/F (L/h) | 0.007 (0.002-0.015) | 0.007 (0.002-0.020) | 2043^a^ (241.9-8308) | 2497^b^ (194.8-8751) | NA | | 2246 (570-8978) | 3563 (669-9725) | NA^f^ | | 1.45 (0.81-4.42) | 1.79 (0.97-13.5) |
| PK parameters are depicted as geometric mean (range), except T_max_ which is depicted as median (range)  ^a^ N=21; AUC_0-∞_ and T_1/2_ could not be reliably estimated for 6 participants because the percentage extrapolated was > 20% | | | | | | | | | | | | |
| ^a^ N=19; AUC_0-∞_ and T_1/2_ could not be reliably estimated for 4 participants because the percentage extrapolated was > 20% | | | | | | | | | | | | |
| ^c^ N=24; One participant was excluded from the analysis due to the development of renal insufficiency between day 15 and day 30, with an increase of ≥ 1.5 times the serum creatinine concentration | | | | | | | | | | | | |
| ^d^ Due to the long T_1/2_ of digoxin AUC_0-24h_ was estimated instead of AUC_0-∞_ | | | | | | | | | | | | |
| ^e^ N=24 AUC_0-∞_ could not be estimated for 1 participant | | | | | | | | | | | | |
| ^f^ AUC_0-∞_ could not be estimated for many participants. AUC_0-last_ (0 to last measurable concentration) was calculated as an alternative. As a result, no T_1/2_ could be calculated either | | | | | | | | | | | | |

| **Table S9. Subgroup analysis of the primary phenotyping metrics of caffeine for smokers and nonsmokers** | | | | |
| --- | --- | --- | --- | --- |
| Caffeine (CYP1A2) | RIF10  (Day 15) | RIF40  (Day 30) | GM RIF40/RIF10 ratio % (90% CI) | CI within 80 – 125% |
|  | h*ug/L (range) | |  |  |
| AUC_0-∞_ total  (n = 25) | 20474   (9989 -75280) | 21574  (7468-75144) | **105 (96 -116)** | Yes |
| AUC_0-∞_ smoking (n = 13) | 14059  (9989-21195) | 13990  (7468-31638) | **100 (87 –114)** | Yes |
| AUC_0-∞_ non-smoking (n = 12) | 30765  (14789 -75280) | 34491   (13343-75144) | **112 (98 – 128)** | No |

| **Table S10. Overview of the CYP genotypes** | | | | | |
| --- | --- | --- | --- | --- | --- |
| *CYP1A2* | *CYP2C9* | *CYP2C19* | *CYP2D6* | *CYP3A4* | *CYP3A5* |
| Genotype, n (%)^a^ | | | | | |
| *1A/*1A 3 (12) | *1/*1 18 (72) | *1/*1 8 (32) | *1/*1 4 (16) | *1/*1 22 (88) | *1/*1 7 (28) |
| *1A/*1F 4 (16) | *1/*2 1 (4) | *1/*17 5 (20) | *1/*17 1 (4) | *1/*22 1 (4) | *1/*3 7 (28) |
| ***1C/*1C^b^ 2 (8)** | *1/*6 1 (4) | *1/*2 4 (16) | *1/*2 3 (12) |  | *1/*6 4 (16) |
| *1F/*1C 9 (36) | *1/*8 2 (8) | *1/*9 5 (20) | *1/*41 1 (4) |  | *1/*7 1 (4) |
| *1F/*1F 3 (12) |  | ***2/*2 1 (4)^b^** | *1/*5 2 (8) |  | *3/*3 3 (12) |
| *1F/*1J 2 (8) |  |  | *17/*17 2 (8) |  | *6/*6 1 (4) |
|  |  |  | *17/*41 1 (4) |  |  |
|  |  |  | *2/*10 1 (4) |  |  |
|  |  |  | *2/*17 1 (4) |  |  |
|  |  |  | *2/*29 1 (4) |  |  |
|  |  |  | *2/*4 2 (8) |  |  |
|  |  |  | *4/*29 1 (4) |  |  |
|  |  |  | *4/*35  (1 allele duplicated) 1 (4) |  |  |
|  |  |  | *5/*29 1 (4) |  |  |
| ^a^ For 2 participants there was insufficient DNA material available to perform this analysis. In addition, the *CYP2D6* and *CYP2C9* genotype could not be determined for 1 participant ^b^ Two participants were classified as possibly having a reduced activity of CYP1A2 (*1C/*1C) and 1 participant was classified as poor metabolizer of CYP2C19 (*2/*2), based on guidelines of the Dutch Pharmacogenetics Working Group (DPWG). | | | | | |

**References**

1. Doroshyenko O, Rokitta D, Zadoyan G, et al. Drug cocktail interaction study on the effect of the orally administered lavender oil preparation silexan on cytochrome P450 enzymes in healthy volunteers. Drug Metab Dispos **2013**; 41(5): 987-93.

2. Zadoyan G, Rokitta D, Klement S, et al. Effect of Ginkgo biloba special extract EGb 761(R) on human cytochrome P450 activity: a cocktail interaction study in healthy volunteers. Eur J Clin Pharmacol **2012**; 68(5): 553-60.

3. Neveling K, Mensenkamp AR, Derks R, et al. BRCA Testing by Single-Molecule Molecular Inversion Probes. Clin Chem **2017**; 63(2): 503-12.

4. Boyle EA, O'Roak BJ, Martin BK, Kumar A, Shendure J. MIPgen: optimized modeling and design of molecular inversion probes for targeted resequencing. Bioinformatics **2014**; 30(18): 2670-2.

5. Langaee T, Hamadeh I, Chapman AB, Gums JG, Johnson JA. A novel simple method for determining CYP2D6 gene copy number and identifying allele(s) with duplication/multiplication. PLoS One **2015**; 10(1): e0113808.

6. Swen JJ, Nijenhuis M, de Boer A, et al. Pharmacogenetics: from bench to byte--an update of guidelines. Clin Pharmacol Ther **2011**; 89(5): 662-73.

7. Gaedigk A, Ingelman-Sundberg M, Miller NA, Leeder JS, Whirl-Carrillo M, Klein TE. The Pharmacogene Variation (PharmVar) Consortium: Incorporation of the Human Cytochrome P450 (CYP) Allele Nomenclature Database. Clin Pharmacol Ther **2018**; 103(3): 399-401.
